# Assessment of Level of Patient Satisfaction after Prostatectomy for Benign Prostatic Hyperplasia in Referral Hospitals in Addis Ababa

**DOI:** 10.4314/ejhs.v30i5.12

**Published:** 2020-09

**Authors:** Andualem Deneke, Mezgeb Gedefe

**Affiliations:** 1 Associate Professor of Surgery, Consultant in Urology, Addis Ababa University, College of Health Sciences, Ethiopia; 2 Urologist, Menilik II Hospital; Addis Ababa Ethiopia

**Keywords:** Prostate, hyperplasia, Prostatectomy, Satisfaction

## Abstract

**Background:**

Benign Prostatic hyperplasia is a very common problem in aging men. TURP comprises 95% of all surgical procedures and is the treatment of choice for prostates sized between 30ml and 80–100ml. Open surgery is the treatment of choice for large glands (bigger than 80–100ml) and for those with associated complications that have indication for open surgery. Many literatures show that the overall patient satisfaction rate and clinical outcome of TURP for BPH are very good. The objective of this study was ‘assessing the level of patient satisfaction after undergoing TURP and TVP for BPH.

**Methods:**

In this research, convenient sampling technique was used. The study design was prospective cohort study. Standardized questioner was prepared in English and later translated into Amharic. Information about demographic characteristics, preoperative data, intraoperative data, and immediate postoperative data were taken while the patient was in the hospital. At the first and the third months after the prostatectomy, patients were inquired on their level of satisfaction about their disease specific satisfaction on the urinary function and their sexual function. We used HCAHPS Measure of Patient Satisfaction Tool, to collect data and analyzed using SPSS version 18.

**Results:**

A total of 89 patients were enrolled in the study among which 65.2% had undergone TURP. The rate of major perioperative complication was found to be low. Over half of the patients had postoperative hospital stay of three or less days. Majority of the patients were satisfied with the care given by the nurses (68.2%) and doctors (84.3%) with relatively higher physicians' care satisfaction level. More than 60% of the patients were highly satisfied with their urinary surgical outcome. With regard to hospital environment, around 60% of the patients reported that they were very satisfied with cleanliness of the rooms, bath rooms and the quietness of the rooms at night.

**Conclusions:**

Both TURP and TVP have high level of patient satisfaction associated with low perioperative major complications. Therefore both can be recommended for patients with clear indications for prostatectomy.

## Introduction

Benign Prostatic Hyperplasia (BPH) is a very common problem in aging men. Based on different literatures, BPH with troublesome lower urinary tract symptoms can affect around 30% of the men aged greater than 65 years (1). According to standard treatment guidelines, BPH can be managed with many treatment modalities based on the level of symptomatology and/or disease progression (e.g. expectant management, medical treatment, minimally invasive treatments, Transurethral Resection of the Prostate (TURP), open prostatectomy etc) (1,2). In the best set ups, TURP comprises 95% of all surgical procedures and is the treatment of choice for prostates sized between 30 ml and 80–100 ml (1). Open surgery is the treatment of choice for large glands (greater than 80–100 ml), and for those with associated complications such as large bladder stones, or bladder diverticula which needs resection (1).

Many literatures show that the overall patient satisfaction rate and clinical outcome of TURP for BPH are very good (see literature review). However, researches that directly assess the level of satisfaction after open prostatectomy are very few. In this study, we tried to show to what level patients were satisfied after the commonly performed procedure (open prostatectomy) in resource limited setups like ours, and that of TURP which is considered to be gold standard for management of BPH.

Patient satisfaction has a variety of definitions in different literatures, but most definitions have ‘underlying patient expectation’ as a common variable. One of the literatures defines it as;

*Patient satisfaction is a cognitive evaluation and an emotional reaction to medical care that is strongly influenced by underlying expectations* (3).

Defining and consequently assessing the level of patient's satisfaction is quite difficult. As such, patient-experience does not simply reflect clinical outcomes or adherence-driven outcomes, rather it seeks to represent a unique encompassing dimension that is challenging to measure objectively (4). The goal of patient satisfaction surveys is to understand from the patient perspective a hospital's/institution's or health system in general specific strengths and weaknesses in order to improve the delivery of care (3).

Patient satisfactions involve multidimensional measures to provide further detail within distinct domains, such as interpersonal manner (communication skill and others) of the provider, technical quality of care, availability, outcomes of care and the physical environment (3). Interpersonal manner, technical quality, accessibility/convenience, and finances have been by far the most commonly measured features of care in patient satisfaction studies (5).

Hospitals with high patient satisfaction provided more efficient care with shorter lengths of stay for surgical patients. These hospitals also had higher surgical process quality, lower surgical readmission rates, and lower surgical mortality rates (6).

Not surprisingly, the improvement in lower urinary tract symptoms is mirrored in the quality of life and patient global impression of improvement scores (7). Most men initially reported that they were “mostly dissatisfied” with their symptoms but this improved, and, at followup, they regarded themselves as “pleased” to “mostly satisfied”. Most men who were asked to compare their current situation to that before the operation, answered “very much better.” No man recorded an unchanged or worse score (7).

Generally, the aim of the treatment of BPH is improving the quality of life (9). R.P. Macdonagh et.al investigated the performance EuroQol (EQ) quality of life measure. The Nottingham Health profile assessed the outcome of TURP for Lower Urinary Tract Symptoms (LUTS) from BPH said that at 12 months after surgery. There were significant improvements in the domains of social interaction, energy, pain, emotional reactions and sleep. The EQ profile also showed a significant improvement in usual activities, mood, and pain/discomfort (9).

Both TURP and open surgery have high profile of efficacy, with marked improvement in International A study done in Japan showed that long-term, more than 10 years, symptomatic improvement was sustained with some deterioration, but quality of life (QoL) remained high (10).

TURP is not only proved to be clinically effective, but also improved patients' QoL and bother symptoms. This was associated with long-term, high patient-rated satisfaction (14).

## Methods

The study design was prospective cohort study. The data was collected at three instances i.e. while the patient was in the ward, at first month, and at third month of the surgery. We took all consecutive patients who had prostatectomy at Menilik II Referral Hospital and Tikur Anbessa Specialized Hospital (TASH) from August 1/2017 to June 15/2018 in Addis Ababa. Convenient sampling technique was used.

All patients who had undergone prostatectomy (either TURP or Transvesical Prostatectomy (TVP)) for BPH by consultant urologist or under the urologist's supervision were included.

Standardized questionnaire was prepared in English and then translated into Amharic and pre-test was done. This questionnaire is a standardized tool, and it was developed by the Consumer Assessment of Healthcare Provider and Systems (CAHPS) Clinical and Group Survey.

Participation on the study was only on voluntary basis. We got informed consent from every patient. We also obtained ethical clearance from Surgical Department Research and Publication Committee of Addis Ababa University, College of Health Sciences (AAU-CHS).

## Results

During the study period, a total of 103 patients had prostatectomy for BPH, 58 from TASH and 45 from Menilik II Hospital. Based on the exclusion criteria, 5 patients (3 for suspicion and/or diagnosis of prostatic cancer, 1 for presence of concomitant bladder tumor, and 1 patient for having contracted bladder) were excluded. There were two referred patients for pneumonia and Acute Respiratory Distress Syndrome (ARDS) requiring mechanical ventilator. There were also two deaths, and 5 patients were lost from follow-up. Finally, we enrolled 89 patients for subsequent assessment of the level of satisfaction after the procedures (56.2% TASH, 43.8% Menilik). Their demographic data is provided in Table 1. The mean age of the patients was 64.8 (ranging from 45 to 92).

**Table 1:** Demographic data of study participants

Variable	Frequency	Percent
Age		
45–65	54	60.7
65–85	32	35.9
Above 85	3	3.4
Marital Status		
Married	70	78.7
Divorced	4	4.5
Widowed	12	13.5
Single	2	2.3
Not registered	1	1.1
Education		
Illiterate	19	21.3
Able to read and write	13	14.6
Primary education	17	19.1
Secondary education	16	18.0
Diploma and above	24	27.0
Employment		
Employed	29	32.6
Private business	9	10.1
Farmer	20	22.5
Pensioned	28	31.5
Other	3	3.3

When we see the presentation of symptoms of the patients, more than 90% of them had multiple lower urinary tract symptoms. History of acute urinary retention was present in 50 patients (60.2%); and 54 patients (62.8 %) had history of catheterization. More than half of these patients stayed with symptoms for more than 1 year before the surgery.

Fifty-eight (65.2 %) of the patients had TURP, and the rest had TVP.

From all patients who had the above procedures, there were six intraoperative bleeding, five during TVP and 1 during TURP. Three Patients required intraoperative and/or post-anesthesia care unit blood transfusion. The intraoperative TURP bleeding forced the surgeon to stop the procedure.

There were no major postoperative complications except 2 patients who had bleeding which required transfusion in the ward. The minor complications found were clot retention (n=4) and wound site infection (n=3). No patient had TURP syndrome. We had two reoperations, the first was re-exploration who developed significant bleeding after TVP and was done immediately after the primary surgery, and the second was re-TURP which was done for incomplete resection because of bleeding which forced the surgeon to terminate the procedure. There were two deaths; one from each type of the surgery. Two patients were also referred to mechanical ventilator after they developed post-operative pneumonia and ARDS.

**Figure 1:**
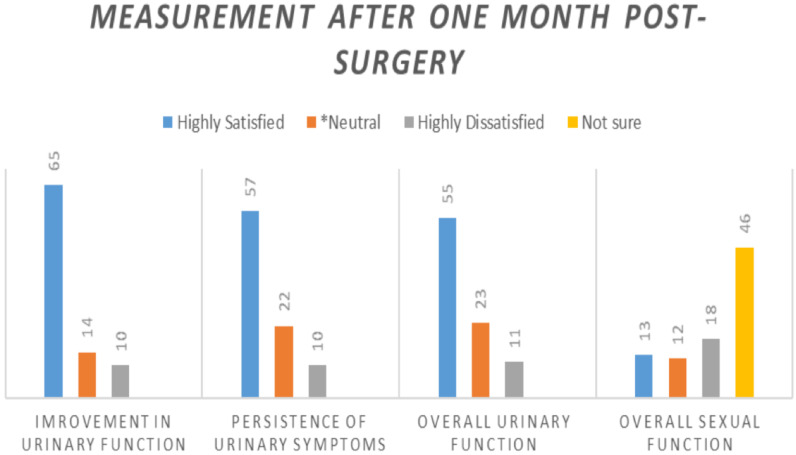
Level of satisfaction from urinary and sexual function after the surgery

**Figure 2:**
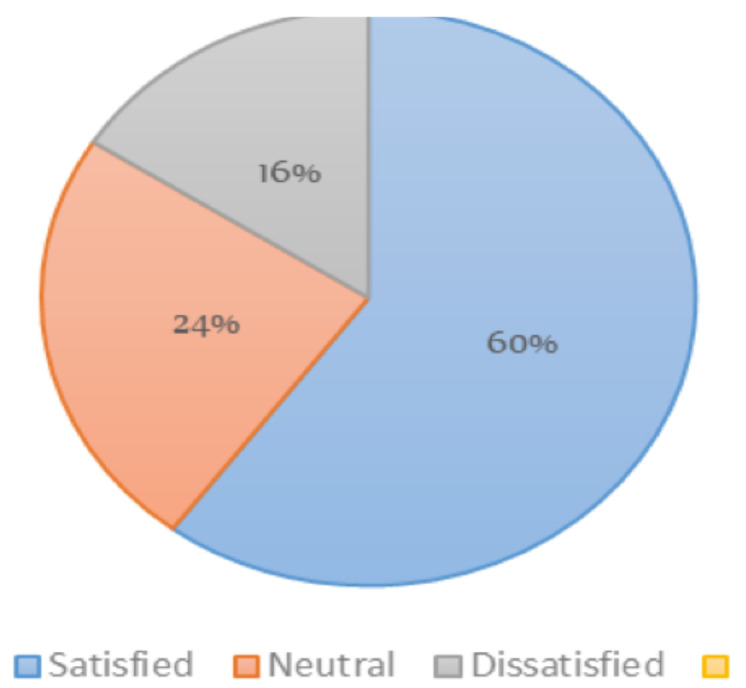
Level of satisfaction from urinary and sexual function after the surgery

The majority of the patients (n=50, 56.1%) were discharged within three days of the postoperative period, and a total of 93.3% of the patients were discharged within seven days of the procedure. About eighty three per cent (84.3%) of these participants wre very satisfied with the doctors' care. About ten per cent (10.2%) and four and half percent (4.5%) of the participants were very dissatisfied with the care given by nurses and doctors respectively. The level of patient satisfactions were assessed for urinary and sexual function and are shown in the figure below (Figures 1 and 2).

In addition to the assessment of urinary and sexual function, patients were also inquired whether they experienced informed or uninformed urinary, sexual, and general surgical complications. The data showed at the end of the first month of the surgery, 54% and 13.6% of the patients had urinary and sexual complications about which they were informed, respectively. Those who experienced untold urinary and sexual complications accounted for 16.7 and 14.7% of the respondents respectively.

For the hospital environment, around 60% of the patients reported they were very satisfied with cleanliness of the rooms and bath rooms and also the quietness of the rooms at night. On the contrary, around 16% were very dissatisfied about the cleanliness of the rooms and bathrooms, and 10% were also very dissatisfied with the quietness of the rooms at night.

Three key questions were asked about the overall satisfaction of the patients on the care provided and the overall assessment of the hospitals. These questions were whether they recommend the same procedures they have undergone to families and close friends. In addition they were asked if recommend the hospitals to their families and close friends. Finally they were asked to grade the hospitals from 1 to 5 (1 being the worst hospital and 5 being the best hospital).

## Discussion

Surgical management of enlarged prostate with lower urinary tract symptoms has different modalities; however, TURP is still considered as the ‘gold standard’.

All of our patients received prophylactic antibiotics. The only significant intraoperative and perioperative complication we found was bleeding requiring transfusion; around 7% of the patients who had TVP had significant bleeding requiring transfusion while none of the patients who had TURP needed blood transfusion. These results may lead to the conclusion that both procedures are safe procedures. This low incidence of complications is even lower than most of the studies reviewed (7,10,11,15).

Satisfaction from surgical outcome of the urinary function is reasonably high and also seems to improve with time as the relative number of patients who were highly satisfied in the first month is increased during third month measurement (Figures 1 and 2). Participants who were dissatisfied with their sexual outcome (from those who had sexual intercourse) were found to be high (around 54.5% at first month and 20% at third month). Most (90.7%) of the patients asked at third month of the post-surgery reported that they recommend the same surgery they had undergone, which can lead us to conclude that both procedures have high degree of satisfaction. With regard to satisfaction level about hospital environment, around two-thirds of the patients were satisfied with the hospitals.

When we consider recommending the surgery, recommending the hospital and rank given to the hospital as a proxy indicators of satisfaction, our results showed outcome related to urinary function and communication skills of healthcare providers to higher relationship.. There was also relationship between level of satisfaction and hospital stay; hospital stay of more than 7 days is shown to be associated with patient dissatisfaction.

Sexual outcome had low relation with the satisfaction with the surgery, and type of surgery was not shown to be related to level of satisfaction. To conclude, whether rate of complications, rate of reoperations, and subsequent procedures related to prostatectomy affect level patient satisfaction or not was difficult because of low incidence of these events.

In conclusion, both TURP and open prostatectomy had high level of patient satisfaction and are also related to significant level of subjective urinary function improvement and have relatively good safety profile. Thus, we recommend both procedures for patients with BPH who are candidates for surgical treatment. Satisfaction about the hospitals was also found to be relatively high.

As both procedures had high level of patient satisfaction and safety profile, more urologists need to be trained in both procedures. The facility for endourology should be available in different parts of the country. General surgery residents should should be well trained with skills in open prostatectomy.

## Figures and Tables

**Figure 3:**
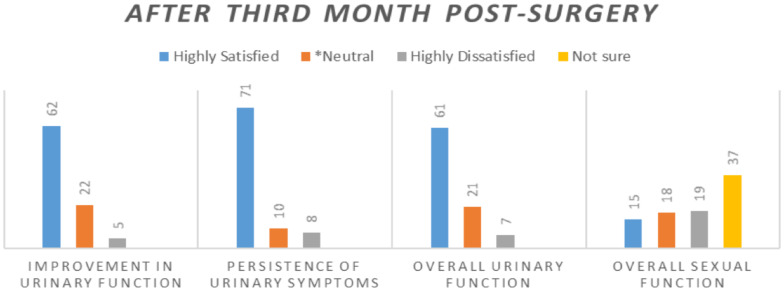
Assessment of satisfaction with cleanliss of rooms and bathrooms

**Table 2:** Preoperative clinical finding

Symptoms	Frequency	Percent
Straining at micturition	87	97.7
Poor urinary stream	84	94.3
Urgency	79	88.7
Frequency	82	92.1
Nocturia (3 times and above)	66	74.1
Urinary incontinence	53	60.9
Acute urinary retention	50	60.2
History of catheterization	54	62.8

**Table 3:** Assessment of overall satisfaction level of participants

Variable	Frequency	Per cent
**Recommend the** **procedure:**		
Yes	78	87.6
No	11	12.4
**Recommend the** **hospital:**		
Yes	56	62.9
Neutral	24	27.0
No	9	10.1
**Rank given to the** **hospital:**		
Among the good hospitals	59	66.3
Neutral	21	23.6
Among the bad/poor hospitals	9	10.1
